# Elevated Troponin Level with Negative Outcome Was Found in Ischemic Stroke

**DOI:** 10.1155/2013/953672

**Published:** 2013-06-13

**Authors:** Buse Hasırcı, Münevver Okay, Dilek Ağırcan, Abdulkadir Koçer

**Affiliations:** Department of Neurology, Istanbul Medeniyet University Medical Faculty, 34720 Istanbul, Turkey

## Abstract

*Background*. Troponin increment is a highly sensitive and specific marker of myocardial necrosis. The reason of high troponin levels in acute stroke is not clear. The aim of this study was to identify the relationships between cardiac troponin-I (cTnI) level and stroke. *Methods*. This study recruited 868 patients who were admitted to Istanbul Medeniyet University due to acute ischemic stroke, and the diagnosis was confirmed by diffusion magnetic resonance imaging. The patients with the causes increasing troponin level were excluded from the study. A total of 239 patients were finally included in the study. Clinics were evaluated by the modified Rankin Scale (mRS) and the National Institutes of Health Stroke Scale (NIHSS). *Results*. Serum level of troponin was higher in ischemic stroke patients with anterior circulation involvement in comparison to posterior involvement or hemorrhagic stroke (*P* < 0.05). Higher troponin levels related to increased stroke scale scores at discharge in ischemic stroke (*P* < 0.05). The level of cTnI was correlated with stroke scale scores at both admission and discharge in posterior stroke patients (*P* < 0.01). *Conclusion*. cTnI is a highly specific and sensitive marker of myocardial damage, and its elevation was associated with more severe neurological deficits in acute ischemic stroke.

## 1. Introduction

Stroke is a leading cause of morbidity and also the third most common cause of death in the developed countries after cancer and ischemic heart disease [1]. The relationship between heart disease and acute stroke was shown previously [2]. It was well known that cardiac enzyme elevation and arrhythmias occurred in some ischemic stroke patients [3–7]. Serum levels of creatinine kinase myocardial fraction (CK-MB), lactate dehydrogenase (LDH), and cardiac troponin T are elevated in myocardial damage [8]. CK-MB levels are elevated not only in myocardial damage, but also in noncardiac conditions [8]. Troponin is a highly sensitive and specific marker of myocardial necrosis in comparison to CK-MB, and it is widely used in the diagnosis of acute myocardial infarction [9–11]. Troponin, a complex of three contractile regulatory proteins, that is, troponin C, T, and I, controls the calcium-mediated interactions between actin and myosin in cardiac and skeletal muscles [12]. Troponin-C is associated with both cardiac and skeletal muscles, but troponin-I and troponin-T are specific to cardiac muscles. Therefore, troponin-C is not used in the diagnosis of myocardial injury [12]. Cardiac troponin-T (cTnT) and cardiac troponin-I (cTnI) are released in cardiomyocytes; cTnT could be released similar to CKMB and LDH-1 in chronically damaged human skeletal muscle without other evidence for myocardial injury [13]. On the other hand, cTnI increment has been reported only after myocardial damage [14]. Troponin is also increased in some acute stroke patients, but the reason of high troponin levels in acute stroke is not clear [14–16]. Autonomic activation after acute ischemic stroke and following in time catecholamine release may induce dysfunction of myocardial tissue [16]. Similarly, cardiac injury might result from sympathoadrenal system activation and autonomic imbalance after stroke affecting cortical areas controlling autonomic function, especially the insular cortex [17, 18].

Autonomic dysfunction resulted in myocytolysis and troponin releasing increased in blood of ischemic stroke patients [14–19]. The blood levels of cTnT were often used in stroke studies [14–16, 19], but serum levels of cTnI which was more specific and reported only after myocardial damage [14] were not much studied previously. To test autonomic dysfunction and its effect on heart, we measured blood levels of cTnI in ischemic and hemorrhagic stroke patients. We also aimed to identify the relationship between serum cTnI and stroke severity. 

## 2. Materials and Methods

All consecutive patients with ischemic or hemorrhagic stroke admitted from January 2010 to December 2012 to the Department of Neurology of the Medeniyet University Hospital, Istanbul, were retrospectively analyzed. Patients were excluded if onset of stroke symptoms was more than 24 h before hospital admission or if no cTnI levels were available. The other exclusion criteria were smoking, drug overuse, or use of any other prophylactic drug such as antidepressants, vitamins, antiepileptics other than topiramate, or daily used anti-inflammatory agents during the preceding 3 months. Patients with atrial fibrillation, cardiac ejection fraction <50%, congestive heart failure, known inflammatory, infectious, or immune disease, diabetes mellitus or glucose intolerance problems, malignancies, or abnormal C reactive protein plasma levels were not included in the study. Baseline characteristics were extracted from medical records and included age, sex, and medical history, which comprised history of hypertension, hypercholesterolemia, diabetes mellitus, previous stroke, coronary artery disease, congestive heart failure, chronic obstructive pulmonary disease, atrial fibrillation, and presence of renal impairment defined as creatinine levels above 1.2 mg/dL. Functional neurological outcome was assessed by using the modified Rankin Scale (mRS) and the National Institutes of Health Stroke Scale (NIHSS) at admission and discharge (including mortality) periods. Stroke was classified as ischemic stroke with involvement of anterior circulation or posterior circulation and hemorrhagic stroke. Involvement area or type of stroke was determined on the basis of imaging findings on computed tomography (64-slice, Siemens AG, Erlangen, Germany) or magnetic resonance imaging (1.5 T, Tim Trio, Siemens AG, Erlangen, Germany). A 4th generation assay (Roche Diagnostics, Mannheim, Germany) was used to measure admission cTnI levels. Values above 0.04 *μ*g/L were considered elevated according to our laboratory ranges. The study protocol was approved by the Ethics Committee of Bezmialem University. 

Statistical analysis was performed using SPSS for Windows, version 11. Clinical features of the patients were evaluated. Mean values and standard deviations were calculated for all variables, but median and minimum-maximum values were used in table because of nonuniform distribution. The Mann-Whitney *U* test and Kruskal-Wallis tests for independent samples and Spearman test were used to analyze differences in median and relationship between groups. The level of statistical significance was set at *P* < 0.05. 

## 3. Results

Eight hundred sixty-eight (*n* = 868) patients were treated at our institution with the diagnosis of ischemic or hemorrhagic stroke. Sixty-seven patients were excluded because symptoms occurred at least 24 h before admission or because of our exclusive criteria mentioned above. Of the remaining 801 patients, admission cTnI values were available in 89% of the patients (*n* = 715). After evaluation with other exclusion criteria, 239 patients were included in our study. 

Elevation of cTnI was identified in 45 patients (18.8%). The patients with increased cTnI were of older age and had more severe strokes, but the differences were not significant as seen in Table 1. NIHSS and mRS scores of patients with and without elevated cTnI at discharge differed (Table 1). The scores were higher in the patients with elevated cTnI. Mean NIHSS score was 15.66 ± 10.94 and 11.62 ± 8.09 *μ*g/L in the patients with and without elevated cTnI at discharge, respectively (*P* = 0.03). Mean mRS score was 4.24 ± 1.32 and 3.72 ± 1.44 *μ*g/L in the patients with and without elevated cTnI at discharge, respectively (*P* = 0.02). 

In comparison of 3 groups of patients, cTnI levels were higher in ischemic stroke patients especially with anterior circulation involvement, and the difference was significant (*P* = 0.04, Table 2). Troponin levels were higher in ischemic stroke patients with anterior circulation than with posterior circulation, and the mean values were 0.08 ± 0.22 and 0.06 ± 0.21 *μ*g/L, respectively (*P* = 0.01). Troponin levels were also higher in ischemic stroke patients with insular cortex involvement in anterior circulation group. A total of 63.87% (*n* = 99) of patients with anterior circulation problem had stroke with insular cortex involvement (Table 1). The percentage of the patients with insular cortex involvement was 64.4% in the group of patients with increased level of cTnI. Twenty-nine percentage of the patients with insular cortex involvement had increased level of cTnI, and this relationship was significant (Table 1, Spearman). The mean values of patients with insular infarcts and without insular infarcts were 0.11 ± 0.29 *μ*g/L and 0.05 ± 0.15, respectively (*P* = 0.001, MWU). In comparison of ischemic stroke patients, the patients with anterior circulation involvement had more severe stroke (Table 2). Although anterior circulation stroke patients had higher level of cTnI, the relationship between cTnI and stroke scales was present at only discharge. cTnI level positively correlated with NIHSS and mRS scores at discharge (*P* = 0.04 and *P* = 0.008, resp.). In contrast, the posterior circulation involvement positively correlated with NIHSS scores at both admission and discharge (*P* = 0.02 and *P* = 0.005, resp.) and mRS scores at both admission and discharge (*P* = 0.001 and *P* = 0.001, resp.). There was not any correlation between cTnI level and stroke scales in the patients with hemorrhagic stroke. 

## 4. Discussion

Relationships between cardiac enzymes and acute stroke are investigated since 1970s [20]. Cardiac enzyme elevation and arrhythmias occurred in some ischemic stroke patients [3–7]. Troponin is a highly sensitive and specific marker of myocardial necrosis in comparison to CK-MB, and it is widely used in the diagnosis of acute myocardial infarction [9–11]. In chronically damaged human skeletal muscle without other evidence for myocardial injury, cTnT levels increased like CK-MB and LDH [13]. On the other hand, cTnI increment has been reported only after myocardial damage [14]. 

 The reason of high troponin levels in acute stroke is not clear [14–16]. Increased intracranial pressure or insular disinhibition leads to marked release of catecholamine by activation of central autonomic network after ischemic stroke, which can induce tachycardia, coronary vasospasm, coronary and peripheral vasoconstriction, and direct myocardial toxicity due to increased intracellular calcium [21]. Song et al. reported that insular cortex involvement has a relationship with elevated serum cTnT and stroke [19]. Principle sensory areas, hypothalamus and paralimbic areas in the orbital, temporopolar, and cingulate cortices, are combined with sensory, autonomic, and limbic functions in the insula. In the literature, it was known that stimulation of insular cortex in epileptic patients indicated that it had cardiac autonomic control [22, 23]. Supporting this concept, insular cortex involvement was shown in %64.4 percentage of people with increased level of cTnI in our study. Also, twenty-nine percentage of the patients with insular cortex involvement had increased level of cTnI, and this relationship was significant as seen in Table 1.

 In stroke patients, troponin elevation is the symptom of acute myocardial infarction or emerge secondary to sympathoadrenal activation. This situation is not clearly understood and leads to dilemma in emergency services. In the present study, our aim was to clarify the relationship between cardiac enzymes and acute stroke. Elevation of cTnI above 0.04 *μ*g/L was identified in 45 patients (18.8%). NIHSS and mRS scores of patients with high cTnI level were higher, and this difference was significant at discharge. cTnI levels were the highest in ischemic stroke patients with anterior circulation. The reason for this could be that the blood supply of insular cortex is coming from anterior circulation and it leads to sympatho-adrenal activation which causes catecholamine release and myocardial damage [17, 18]. The blood levels of cTnT were often used in stroke studies [14–16, 19], but serum levels of cTnI were not much studied previously. Di Angelantonio et al. reported that cTnI levels highly correlated with mortality, and they suggested that cTnI positivity might be an independent prognostic predictor in ischemia [21]. They found that 5.5% of the patients had higher cTnI level (0.40 ng/mL), but we have found that more patients (18.8% of the patients) had higher cTnI levels [21]. Our results are more valuable, because the patient group in our study are more isolated because of rigid inclusion criteria. 

Neuroimaging studies in humans identified that dorsal and subgenual regions of the anterior cingulate cortex, insular cortex, and to a lesser extent the amygdala and basal ganglia are main locations involved in cardiac control [20]. In addition, there is now substantial evidence that the brainstem affects cardiac electrophysiology and arrhythmia. The periaqueductal grey and parabrachial nucleus formulate descending drive to the heart by the integration of afferent baroreceptor and mechanoreceptor information in the brainstem [11]. The autonomic nervous system which is located in the medulla oblongata in the lower brainstem, has an important role in the genesis, maintenance, and interruption of ventricular arrhythmias [24]. In most instances, sympathetic activation precipitates or enhances ventricular arrhythmias [25, 26]. Sympathetic nerve fibers are located subepicardially and travel along the routes of the major coronary arteries. A lesion of the heart produced by infarct or fibrosis can result in denervation of otherwise normal myocardium by interruption of neural axons traveling through the lesion [27]. In short, autonomic nervous system which is located in brainstem has a role on cardiac control. Its interruption due to posterior circulation stroke leads to cardiac arrhythmias, myocardial infarction which leads to myocardial damage, and troponin increment. Supporting this knowledge, we found that cTnI positively correlated with scores at both admission and discharge in patients with posterior circulation involvement. 

 Posterior hypothalamus and midbrain reticular formation stimulation cause ECG changes similar to those following subarachnoid hemorrhage [28]. Most studies revealed hypothalamus as the primary center of cardiac dysfunction in the patients with subarachnoid or intracerebral hemorrhages [29, 30]. In a newly published material, Chung et al. showed that elevated troponin levels in patients with intracerebral hemorrhage were related to poor outcomes [31]. However, there was not any correlation between cTnI level and stroke scales in the patients with hemorrhagic stroke in our study. Additionally, cTnI levels were lower in comparison to ischemic stroke patients. Further studies are needed to clarify the clinical implications of serum cTnI levels in patients with intracerebral hemorrhage. 

Its retrospective nature and short follow-up period were limitations of the present study. Despite these limitations, we believe that the findings of this study will improve the understanding of the relationship between cardiac enzymes with ischemic stroke patients and hemorrhagic stroke patients and also its effect on prognosis. The present study showed that elevated cTnI levels in patients with acute stroke were related to poor outcomes. Although it was known that serum levels of cTnI was more specific to myocardial damage, troponin levels were also higher in ischemic stroke patients and positively correlated with outcome scores. As a conclusion, stroke is not problem itself. Stroke-related complications such as infections or wounds are well known, but cardiac dysrhythmia or myocardial infarction titles were waiting to be evaluated. Maybe it will be better that routine tests in emergency services should include cTnI. Long-term followup and serial serum cTnI evaluation from a large prospective study may illuminate the clinical impact of elevated serum cTnI levels in stroke patients. 

## Figures and Tables

**Table 1:** Clinical characteristics of patients with and without elevated cTnI.

Variable	Troponin elevation		*P* value^*α*^
	Yes (*n* = 45)	No (*n* = 194)	
Age (year) median* (Min–Max)**	78 (*R*: 42–98)	73 (*R*: 24–93)	0.08
NIHSS at hospitalization median (Min–Max)	13 (*R*: 1–19)	12 (*R*: 0–19)	0.34
NIHSS at discharge median (Min–Max)	12 (*R*: 0–36)	11 (*R*: 1–36)	*0.03 *
mRS at hospitalization median (Min–Max)	4 (*R*: 1–5)	4 (*R*: 0–12)	0.09
mRS at discharge median (Min–Max)	4 (*R*: 1–6)	4 (*R*: 0–11)	*0.02 *
Duration of hospitalization (day) median (Min–Max)	7 (*R*: 1–39)	6 (*R*: 1–34)	0.95
Ischemic stroke patients (*n*)		
Anterior circulation	32	123
Posterior circulation	8	49	0.55
Hemorrhagic stroke patients (*n*)	5	22	
Insular cortex involvement	29	70	*0.001* ^β^
Gender (female/male)	28/17	98/96	0.15

*Median, **range (Min–Max), ^*α*^Mann-Whitney *U* test, ^β^Spearman correlation.

**Table 2:** Clinical characteristics of patients with ischemic and hemorrhagic stroke patients.

Variable	Stroke type				*P* ^β^ value
	Ischemic			Hemorrhagic (*N* = 27)	
	Anterior circulation (*N* = 155)	Posterior circulation (*N* = 57)	*P* ^*α*^ value		
Age (year)	76* (24–93)**	71 (30–98)	0.02	73 (51–92)	0.05
cTnI level (*μ*g/l)	0.01 (0–1.93)	0.01 (0–1.41)	0.01	0.01 (0–1.25)	0.04
NIHSS at hospitalization	13 (1–19)	7 (0–18)	0.00	12 (3–17)	0.00
NIHSS at discharge	12 (1–36)	6 (0–36)	0.00	10 (3–36)	0.00
mRS at hospitalization	4 (1–12)	3 (0–5)	0.00	4 (1–5)	0.00
mRS at discharge	4 (1–11)	3 (0–6)	0.00	4 (1–6)	0.00
Duration of hospitalization (day)	7 (1–34)	6 (1–14)	0.08	8 (1–39)	0.21

*Median, **range (Min–Max), ^*α*^Mann-Whitney *U* test, ^β^Kruskal-Wallis test.
